# In Memoriam: Dr. Vijay Davalbhakta (1938–2023)

**DOI:** 10.1055/s-0045-1811240

**Published:** 2025-08-29

**Authors:** Ashish Davalbhakta

**Affiliations:** 1 Department of Aesthetic Plastic Surgery, Advanced Aesthetics & Aesthetics Medispa, Affiliated to Maharastra University of Health Sciences, Pune, Maharashtra, India

**Fig. 1:**
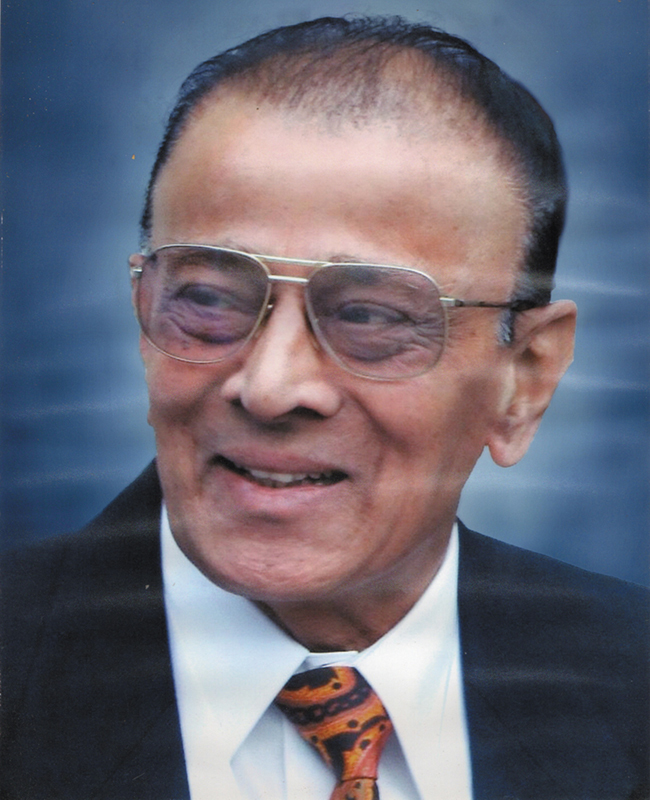
Dr. Vijay Davalbhakta.

Dr. Vijay Davalbhakta was born on July 7, 1938 and passed away on November 7, 2023, at the age of 85 ( Fig. 1 ). A life member of Association of Plastic Surgeons of India (APSI) since 1982, he lived a remarkable life that left a deep imprint on the lives of his patients, colleagues, students, and family. As his son, I had the privilege of observing his life's work from close quarters—a journey that was as inspiring as it was humbling.

He completed his MBBS from B. J. Medical College, Ahmedabad, and pursued general surgical training at the prestigious J. J. Hospital and St. George's Hospital, Mumbai. It was at J. J. Hospital that he came under the influence of Dr. N. H. Antia, a doyen of Indian Plastic Surgery, who inspired him to take up the specialty as his calling.

**Fig. 2:**
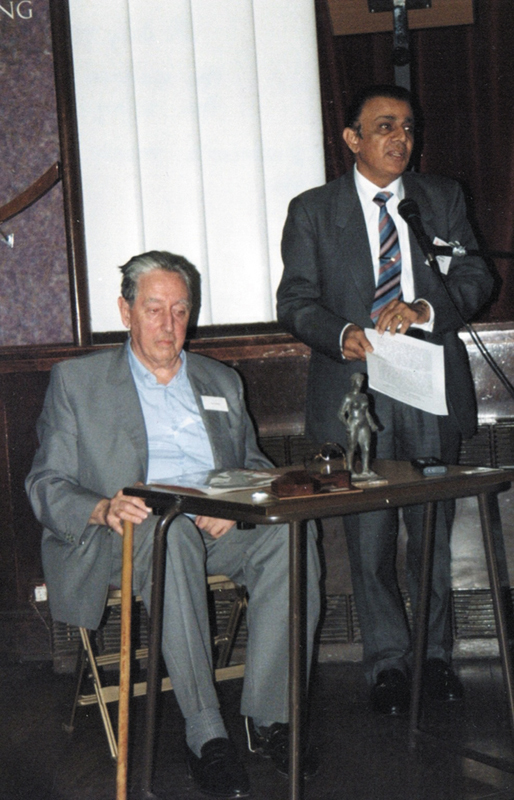
With Mr. Tony Wallace, Broomfield Hospital at the 25th Anniversary.

Aspiring to gain deeper expertise, he travelled to the United Kingdom in 1969. At first, it was a struggle to find a training opportunity in Plastic Surgery. When he learned that Dr. Antia was visiting London, he met him and shared his challenges. Dr. Antia graciously spoke to Mr. Dawson, the then Secretary of the British Association of Plastic Surgeons, who in turn contacted Dr. Antony Wallace of the St. Andrew's Centre for Plastic Surgery at Broomfield Hospital. Although the interviews had concluded, Dr. Wallace agreed to see him and, impressed with his resolve and credentials, offered him a position ( Fig. 2 ).

That marked the beginning of his illustrious career in Plastic Surgery. He worked under Dr. Wallace at St. Andrew's Centre for Plastic Surgery, Broomfield Hospital, United Kingdom, for over a year and a half and then continued his training at Stoke Mandeville Hospital, Aylesbury, under Mr. Bruce Bailey. Over 3 years, he gained thorough grounding in Plastic, Reconstructive, and Burn Surgery.

When it was time to return to India, Dr. Antia, true to his philosophy of community service and local empowerment, once again advised him. “Why not your hometown, Jalgaon?” he said. And so, in 1972, Dr. Vijay Davalbhakta took over the reins of his father's private hospital, becoming the first plastic surgeon in Northern Maharashtra, like his father before him, who was the first qualified General Surgeon in the area. He brought advanced reconstructive procedures to a region that had long lacked such services. He served a vast region where there had been little access to specialist care for congenital anomalies, trauma, cancer reconstruction, and burn injuries. His practice flourished—and with quiet dignity, he built an institution.

**Fig. 3:**
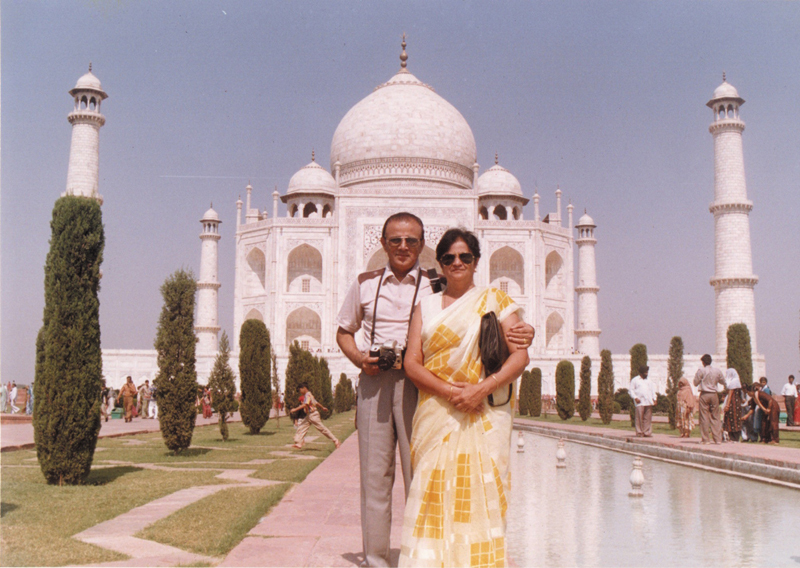
His partner during plastic surgery and in life.

I grew up watching him operate and deliver wondrous results. My mother, Dr. Trupta Davalbhakta, was his perpetual anesthetist ( Fig. 3 ). They worked seamlessly as a team—surgery in the mornings, completed before I returned from school, followed by bustling outpatient clinics in the evenings. The patient's ability to pay used to become the accepted fee rather than the standard rate card. One particular noteworthy feature of his practice was that the postoperative stay in the hospital was almost free, till the wound was absolutely dry and healed. I have seen patients staying admitted for weeks if not months, and the patients never complained as there was no additional charge. Their dedication and partnership set the foundation for my own career in Plastic Surgery.

We attended almost every national plastic surgery conference together—even during my undergraduate years. His wife, Dr. Trupta Davalbhakta, accompanied him to each and every professional engagement. They were truly a team—in work and in life.

**Fig. 4:**
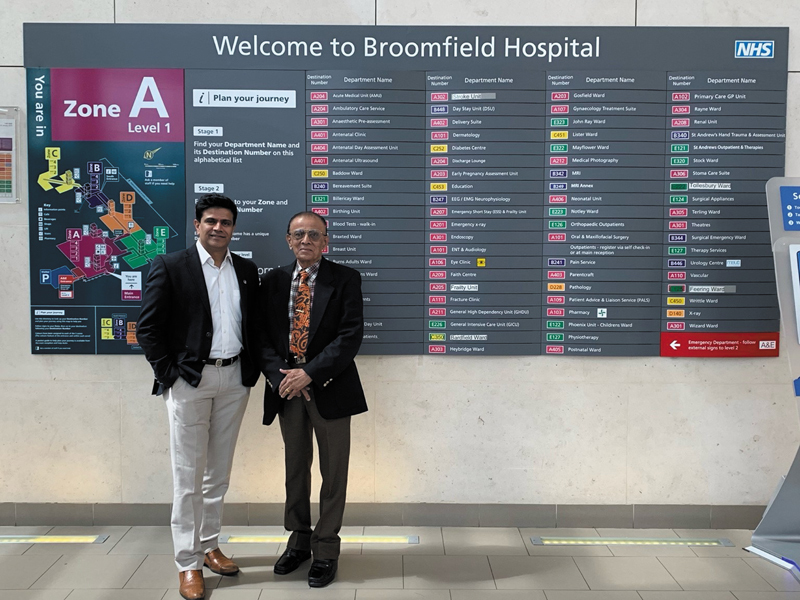
Father and son duo, who worked in St. Andrews Centre for Plastic Surgery, Broomfield Hospital, United Kingdom.

He was overjoyed when I got my first NHS SHO (National Health Service Senior House Officer) job at the very same St. Andrew's Centre for Plastic Surgery at Broomfield Hospital. He would proudly tell people that the Davalbhaktas were the only father–son duo to have worked in that unit ( Fig. 4 ).

**Fig. 5:**
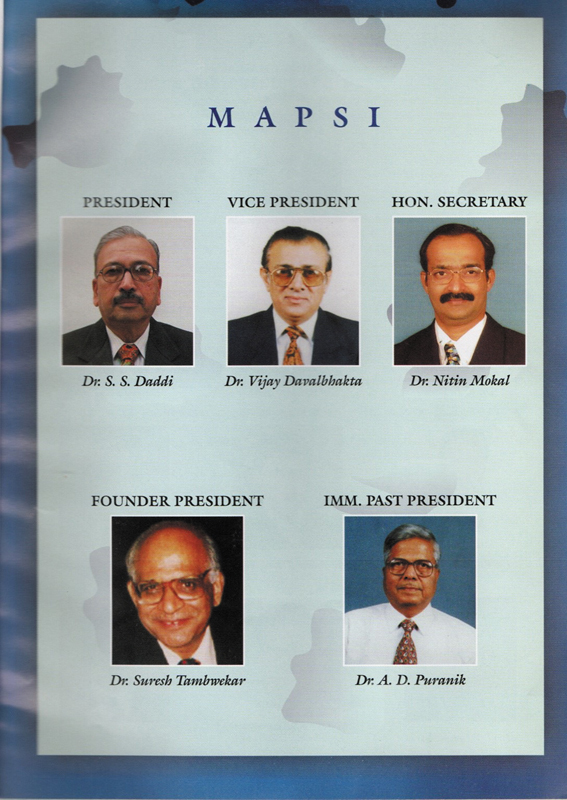
MAPSI Executive Body, with Dr. Vijay Davalbhakta.

**Fig. 6:**
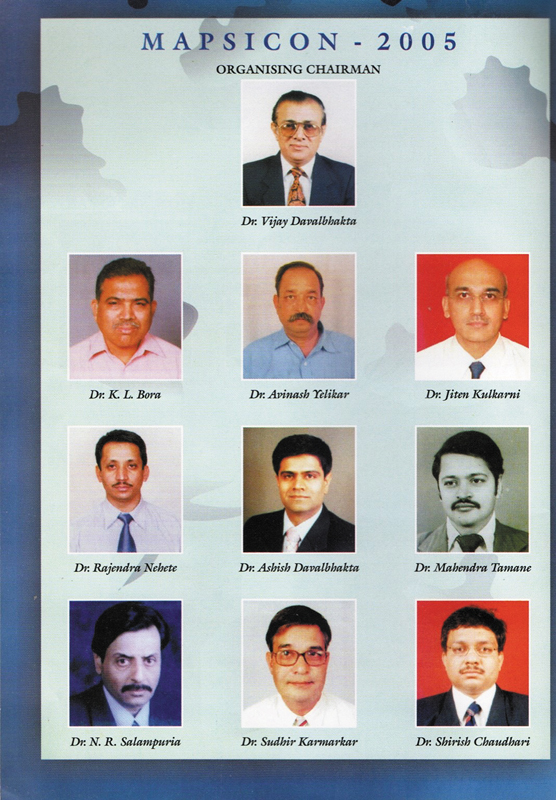
MAPSICON Organizing Committee.

**Fig. 7:**
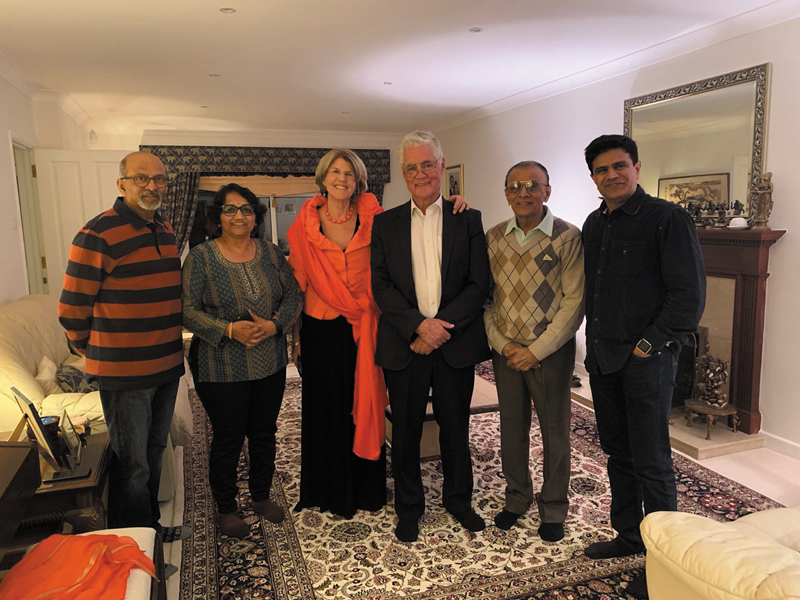
With Mr. David Elliot and Niri Niranjan—my bosses, who became his close friends.

**Fig. 8:**
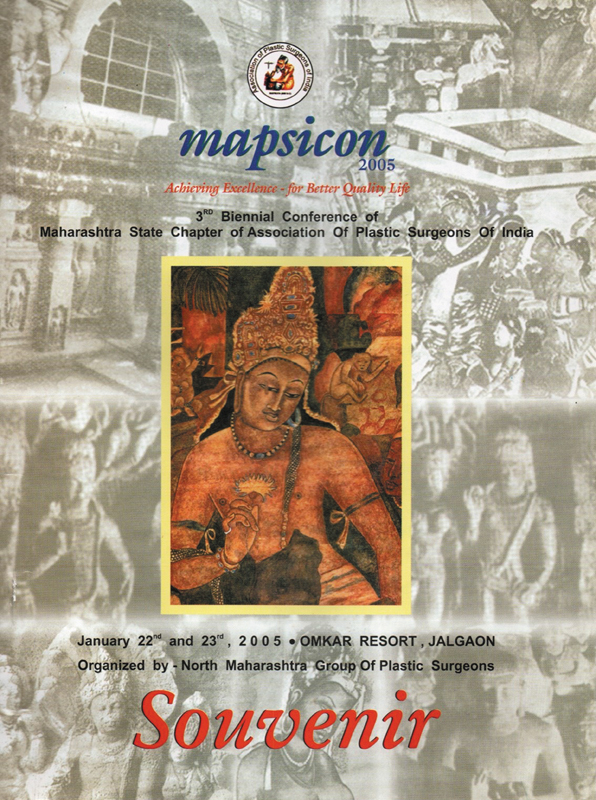
Souvenir from MAPSICON conference, Jalgaon.

His clinical expertise was widely respected. Dr. Suresh Tambwekar, a pioneering leader in Maharashtra Plastic Surgery, recommended him as the fourth President of the Maharashtra State Chapter of the Association of Plastic Surgeons of India (MAPSI), a role he accepted with honor. Though initially concerned about hosting a state-level conference in a smaller town like Jalgaon, he went on to organize one of the most memorable meetings ( Figs. 5 and 6 ). The conference was attended by stalwarts such as Dr. Goleria, Dr. R. L. Thatte, Dr. Ashok Gupta, Dr. Tambwekar, Dr. Mukund Thatte, Dr. Raja Sabapathy, and Dr. Shrirang Pandit, among many others. Through his United Kingdom connections, he even brought in two international speakers—Mr. David Elliot and Mr. Niri Niranjan—a first for a MAPSI conference ( Fig. 7 ). The conference was greatly appreciated for not only its high level of scientific deliberations, but also for the homely and warm hospitality ( Fig. 8 ).

He continued to be a guiding light and a fatherly figure for the Maharashtra Association of Plastic Surgeons and was honored with the post of Trustee of the MAPS, which he responsibly held from 2013 to 2019.

**Fig. 9:**
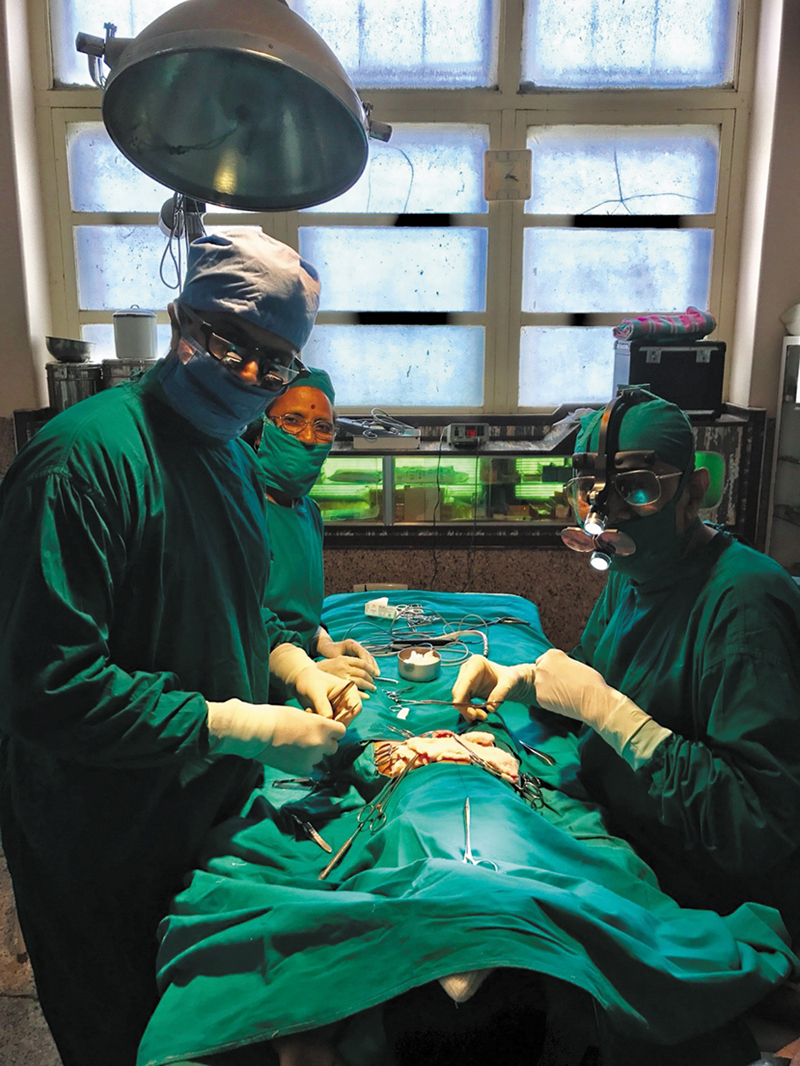
Assisting Dr. Vijay Davalbhakta at one of his hypospadias operations.

He remained humble to the core. Never ostentatious, always approachable. He was studious even in his later years, attending APSICON (Annual Conference of the Association of Plastic Surgeons of India) and AESURG meetings with me. After I began my practice in Pune, I routinely visited our hospital in Jalgaon. He would assist me in aesthetic and reconstructive surgeries, while I assisted him in his forte—especially hypospadias repair ( Fig. 9 ). He had developed a unique two/three-stage Mathieu flip-flap technique and was proud of his zero fistula rate. He was so confident of his watertight closures that he never kept a catheter postoperatively—patients would void normally the same evening.

Another area of his expertise was the use of modified rhomboid flaps for facial defects. Despite the challenges of working with dark-pigmented skin in central Maharashtra, his results were aesthetically pleasing and reliable. His lectures on these topics were clear, evidence-based, and enriched with decades of experience.

I was fortunate to inherit a vast number of secondary cleft lip nose cases. All the patients who had their primary cleft lips and palates done by him, years earlier, were revisiting for nose improvements. He would thoroughly enjoy assisting me throughout the surgery and I could see a deep sense of satisfaction on seeing complete rehabilitation of his patient.

**Fig. 10:**
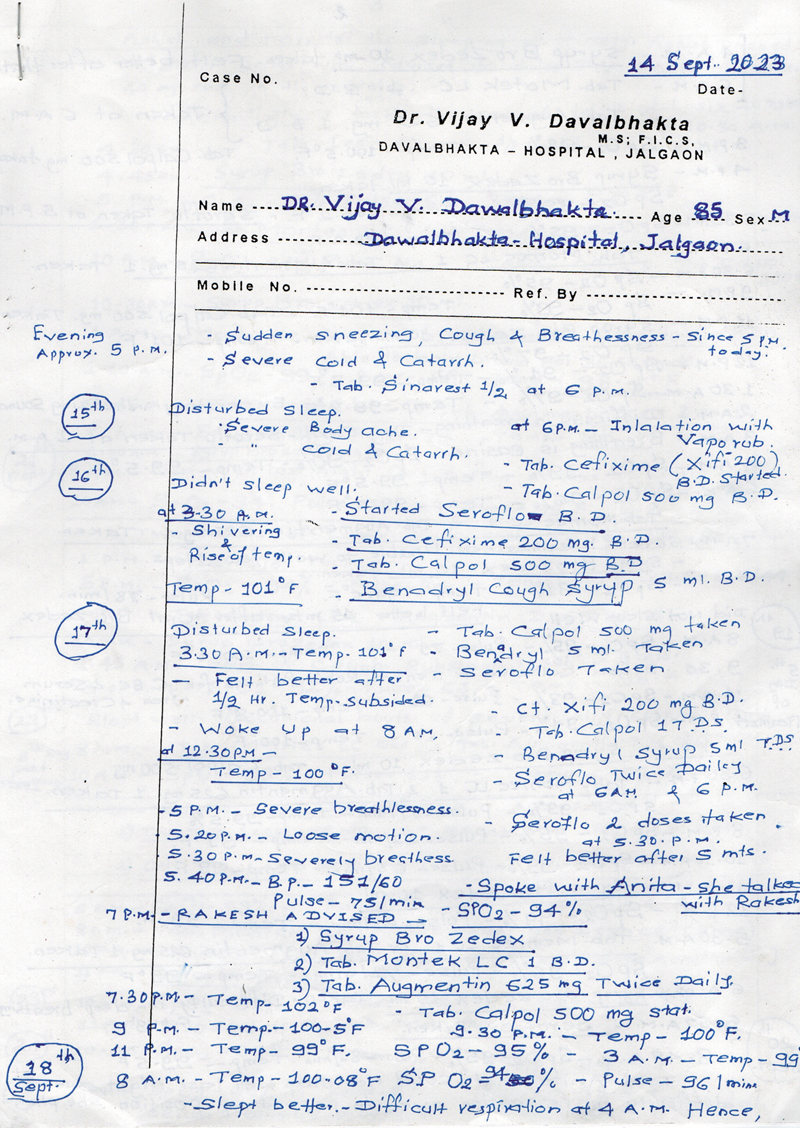
Meticulous note keeping.

He was born to be a Plastic Surgeon—his meticulous note-keeping and documentation were legendary. His nurses could retrieve records from 50 years ago with clear typewriter-like handwriting detailing every aspect of the case ( Fig. 10 ). His study shelves were filled with transparency slides—pre-op, intra-op, and post-op images—viewable only via a slide projector or slide viewer. This meticulous record keeping was definitely a first for his generation.

He believed deeply in giving back to society. He served as the President of the Lana Shikshan Prasarak Mandal, which ran his alma mater. He was an active Rotarian, who served Rotary as its President and as a Group Study Exchange leader. He was actively involved in health camps and immunization programs through Rotary. Most of his professional life, he offered his services as an honorary plastic surgeon at the Civil Hospital in Jalgaon, performing countless surgeries free of charge and training interns and residents. His standing in Jalgaon was more than that of a doctor—he was a respected dignitary.

**Fig. 11:**
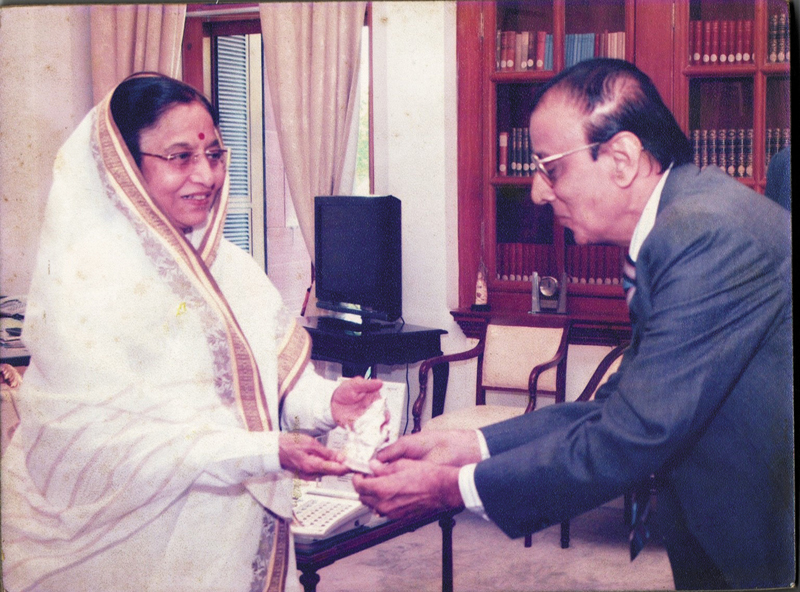
Gifting a silver Ganesha to President Hon. Pratibhatai Patil.

One particularly proud moment for him was when Dr. Rajiv Ahuja, the President of APSI, asked him to facilitate a meeting with Mrs. Pratibhatai Patil, the President of India. Through his close contacts, he arranged the audience, and the photograph of him gifting a silver Ganesha to the President remains a treasured memory ( Fig. 11 ).

**Fig. 12:**
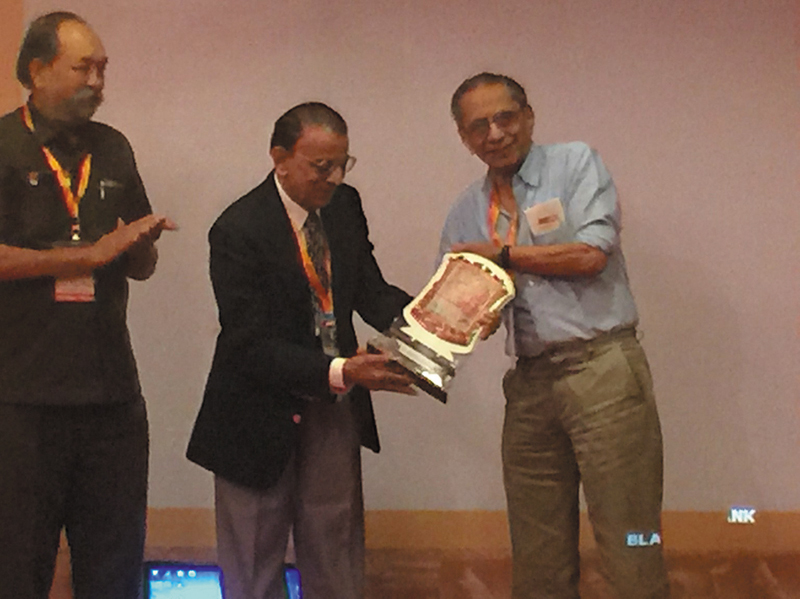
Dr. Vijay Davalbhakta felicitating Dr. RL Thatte at Kolhapur MAPSCON.

His friend and colleague, Dr. R. L. Thatte, summed up his spirit beautifully:

“In all my life I have never met a man more gentlemanly than Dr. Vijay Davalbhakta. Always well-dressed, polite to a fault, ever smiling, willing to listen to others and genuinely interested in other people's problems. But behind that exterior was an absolute first-class plastic surgeon whose work in the hinterland of Maharashtra was equal to, or even better than, similar work in urban academic circles. What is more, his presentations of his work were impeccably and beautifully delivered. In a world now driven by technology and forever in a hurry, his memory will always soothe me with sheer old-world charm” ( Fig. 12 ).

Dr. Mukund Thatte, past president of APSI, reminisced “I first met Dr. Vijay Davalbhakta in APSICON 1986. He struck me as a calm and composed person. Soft spoken yet very good at his work. I kept meeting him in various national and state meetings. He was the president of the Maharashtra state association and organized a wonderful meeting in Jalgaon. One of the highlights was a panel on scar management where scars were shown as a pre-op picture and the panel along with the delegates discussed options. He then showed results of what he had done and they were amazing. His enduring image in my mind is of a cultured competent and soft-spoken individual.

Dr. Medha Bhave, another senior Plastic Surgeon from Maharashtra had these memories of Dr. Vijay Davalbhakta “My late grandfather, Dr. G. S. Khair, a well-known educationist, had mentioned several times about Dr. Davalbhakta from Jalgaon. He used to conduct a Dant Yadnya. It was meant for poor people who could not afford dental extractions and often landed with quacks. He used to perform extractions very deftly and painlessly.

When I met Dr. Vijay Davalbhakta, after asking about Dant Yadnya, he told me he knew my grandfather very well through his dentist uncle who used to organize it. We bonded instantly. He had a fatherly aura with a calm, satisfied attitude. His problem solving was based on quick analysis of the situation, and innate honesty, with which he resolved some association disputes.

He enthusiastically attended meetings sitting on front seat, occasionally discussing his points of disagreement. He would always ask me where and when I would be talking and made it a point not only to attend but later come and compliment.

We always used to have customary photo together.

Listening to the problems he faced in practice was real education. His humanitarian, selfless yet scientific and logical approach to life always impressed me.

He maintained himself well and worked till long. Moving to periphery in those days and practicing subspeciality must have been full of challenges. Yet he never once lost his soft charm that I will always miss.”

He worked till the very end as he did not believe in retiring. He lived a life of purpose. He worked with devotion. And he leaves behind a legacy of humility, healing, and honor.

